# An MRI evaluation of white matter involvement in paradigmatic forms of spastic ataxia: results from the multi-center PROSPAX study

**DOI:** 10.1007/s00415-024-12505-y

**Published:** 2024-06-16

**Authors:** Alessandra Scaravilli, Ilaria Gabusi, Gaia Mari, Matteo Battocchio, Sara Bosticardo, Simona Schiavi, Benjamin Bender, Christoph Kessler, Bernard Brais, Roberta La Piana, Bart P. van de Warrenburg, Mirco Cosottini, Dagmar Timmann, PROSPAX Consortium, Alessandro Daducci, Rebecca Schüle, Matthis Synofzik, Filippo Maria Santorelli, Sirio Cocozza

**Affiliations:** 1 Department of Advanced Biomedical Sciences, University of Naples “Federico II”, Via Pansini 5, 80131 Naples, Italy; 2 Department of Computer Science, Diffusion Imaging and Connectivity Estimation (DICE) Lab, University of Verona, Verona, Italy; 3 Department of Diagnostic and Interventional Neuroradiology, University of Tübingen, Tübingen, Germany; 4 Center for Neurology and Hertie Institute for Clinical Brain Research, University of Tübingen, Tübingen, Germany; 5 Department of Neurology and Neurosurgery, Montreal Neurological Institute, McGill University, Montreal, Canada; 6 Department of Diagnostic Radiology, McGill University, Montreal, Canada; 7 Department of Neurology, Donders Institute for Brain, Cognition, and Behaviour, Radboud University Medical Center, Nijmegen, The Netherlands; 8 Department of Translational Research on New Technologies in Medicine and Surgery, University of Pisa, Pisa, Italy; 9 Department of Neurology and Center for Translational Neuro- and Behavioral Sciences (C-TNBS), Essen University Hospital, Essen, Germany; 10 German Center for Neurodegenerative Diseases (DZNE), Tübingen, Germany; 11 Division of Neurodegenerative Diseases, Department of Neurology, Heidelberg University Hospital and Faculty of Medicine, Heidelberg, Germany; 12 Division Translational Genomics of Neurodegenerative Diseases, Center for Neurology and Hertie Institute for Clinical Brain Research, University of Tübingen, Tübingen, Germany; 13 Department of Molecular Medicine, IRCCS Stella Maris Foundation, Pisa, Italy

**Keywords:** ARSACS, SPG7, Magnetic resonance imaging, Diffusion tensor imaging, Ataxia

## Abstract

**Background:**

Autosomal Recessive Spastic Ataxia of Charlevoix-Saguenay (ARSACS) and Spastic Paraplegia Type 7 (SPG7) are paradigmatic spastic ataxias (SPAX) with suggested white matter (WM) involvement. Aim of this work was to thoroughly disentangle the degree of WM involvement in these conditions, evaluating both macrostructure and microstructure via the analysis of diffusion MRI (dMRI) data.

**Material and methods:**

In this multi-center prospective study, ARSACS and SPG7 patients and Healthy Controls (HC) were enrolled, all undergoing a standardized dMRI protocol and a clinimetrics evaluation including the Scale for the Assessment and Rating of Ataxia (SARA). Differences in terms of WM volume or global microstructural WM metrics were probed, as well as the possible occurrence of a spatially defined microstructural WM involvement via voxel-wise analyses, and its correlation with patients’ clinical status.

**Results:**

Data of 37 ARSACS (M/F = 21/16; 33.4 ± 12.4 years), 37 SPG7 (M/F = 24/13; 55.7 ± 10.7 years), and 29 HC (M/F = 13/16; 42.1 ± 17.2 years) were analyzed. While in SPG7, only a mild mean microstructural damage was found compared to HC, ARSACS patients present a severe WM involvement, with a reduced global volume (*p* < 0.001), an alteration of all microstructural metrics (all with *p* < 0.001), without a spatially defined pattern of damage but with a prominent involvement of commissural fibers. Finally, in ARSACS, a correlation between microstructural damage and SARA scores was found (*p* = 0.004).

**Conclusion:**

In ARSACS, but not SPG7 patients, we observed a complex and multi-faced involvement of brain WM, with a clinically meaningful widespread loss of axonal and dendritic integrity, secondary demyelination and, overall, a reduction in cellularity and volume.

**Supplementary Information:**

The online version contains supplementary material available at 10.1007/s00415-024-12505-y.

## Introduction

Spastic ataxia (SPAX) represents a clinical phenotype characterized by the presence of cerebellar ataxia along with spasticity and other pyramidal features [1, 2]. This phenotype is associated with a heterogeneous group of predominantly genetic conditions [3]. Among these, Autosomal Recessive Spastic Ataxia of Charlevoix-Saguenay (ARSACS, MIM #270550) and Spastic Paraplegia Type 7 (SPG7, MIM #607259) represent some of the most common forms of autosomal recessive cerebellar ataxias reported in literature, and might, therefore, be used as good models to understand more of the pathophysiological mechanisms behind the development of brain damage in genetically determined SPAX [2, 4].

In this light, magnetic resonance imaging (MRI) plays an unquestionable role in the evaluation of patients with neurodegenerative disorders, including SPAX patients, not only for clinical purposes but also by providing quantitative, reliable, and in vivo information on neuronal damage [5, 6] during the disease course. Among all the sequences available, diffusion MRI (dMRI) is a quantitative technique able to capture subtle but meaningful changes occurring in several conditions, from neurodevelopmental to neurodegenerative, as well as psychiatric or neuroinflammatory disorders [7–12]. This method, sensitive to water diffusion characteristics in brain tissue, represent a marker of white matter (WM) microstructural integrity [13], with different parameters that can be extracted from a single sequence but that can provide simultaneously complementary information about neuronal integrity [14, 15]. Some scattered descriptions about a certain degree of WM involvement, using dMRI, have been previously reported in both ARSACS and SPG7 patients [16–19]. Nevertheless, this information is derived from studies with very small sample size (or even single case reports in SPG7) and relatively outdated processing methods, therefore significantly limiting our comprehension of possible, more profound, pathophysiological changes that might occur in the WM of patients with these disorders.

In this study, we aimed to expand the current knowledge about WM involvement in SPAX patients leveraging the largest standardized dataset of ARSACS and SPG7 patients available to date, collected within the PROSPAX consortium, which is an international multi-center collaborative research project on SPAX [20]. The main aim of this work was to thoroughly disentangle the degree of WM involvement in these two conditions, through a “top to bottom” approach from macrostructure to microstructure. Specifically, we aimed to assess if there were differences between ARSACS, SPG7 patients, and Healthy Controls (HC) in terms of: (1) WM volume, (2) global microstructural WM metrics, and (3) if spatially defined differences of microstructural WM metrics are present. Finally, (4) we correlated microstructural WM changes and detailed clinimetrics to derive more precise understanding of imaging patterns in SPAX.

## Materials and methods

### Compliance with ethical standards

Data analyzed in this study were acquired within the multi-center project PROSPAX (“An integrated multi-modal progression chart in spastic ataxias”-ClinicalTrials.gov no: NCT04297891). The study was approved by local Ethics Committee of each center and written informed consent was obtained from each participant.

### Participants

**Fig. 1:**
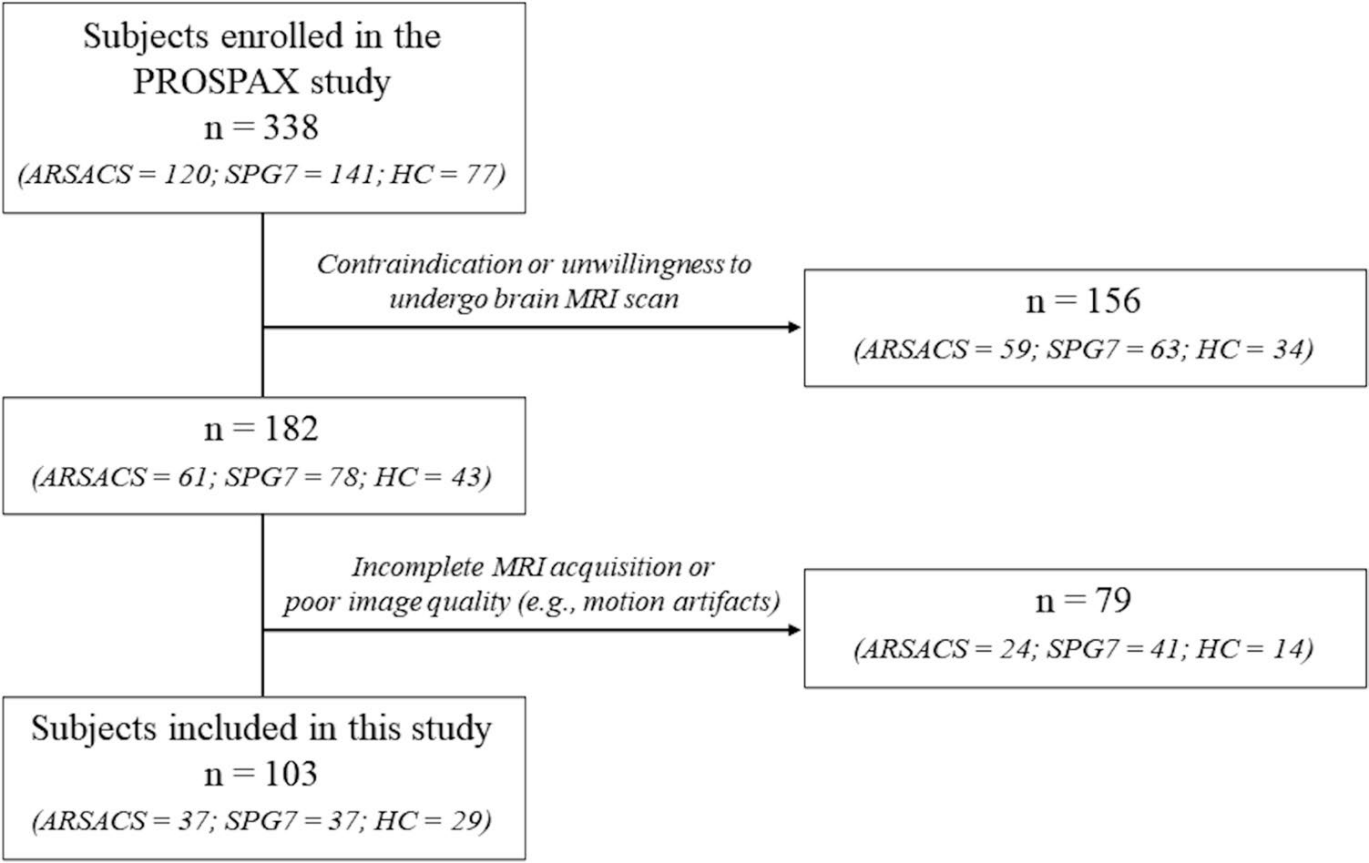
Flowchart showing the procedure followed to reach the final number of subjects analyzed in this study

In this multi-center prospective study performed in 8 centers from 6 countries from January 2021 to October 2022, 120 ARSACS patients and 141 SPG7 genetically confirmed patients, along with 77 Healthy Controls (HC) with no history of neurological or psychiatric disorders, were enrolled. All subjects with contraindication or unwillingness to undergo a brain MRI scan were then excluded from this study. After the application of this first exclusion criteria, subjects with an incomplete MRI acquisition (namely, without a T1-weighted volume and a dMRI sequence acquired within the same MR session) or with a poor quality of images (e.g., due to motion artifacts or data corruption) were then further excluded from the study (Fig. 1). For the assessment of disease severity, a neurologic examination within 1 month from the MRI acquisition was performed for each subject via the Scale for the Assessment and Rating of Ataxia (SARA) [21], the Spastic Paraplegia Rating Scale (SPRS) [22] (with the record of the corresponding “functional mobility” items from 1 to 6—fSPRS) and the Friedreich’s Ataxia Rating Scale (FARS) [23]. Furthermore, the Montreal Cognitive Assessment (MoCA) test was also administered to provide a basic cognitive assessment of these patients [24].

### MRI data acquisition

All sites acquired a standardized harmonized dMRI protocol on a 3 T scanner with the following parameters: TR = 4200 ms; TE = 90 ms; flip angle = 90°; voxel size = 2 × 2 × 2 mm^3^ with 3 directions at *b* = 300 s/mm^2^, 6 directions at *b* = 700 s/mm^2^, 32 directions at *b* = 1000 s/mm^2^, 50 directions at *b* = 2000 s/mm^2^ in addition to 7 directions at *b* = 0 s/mm^2^; GRAPPA acceleration factor = 2; phase encoding = AP; bandwidth = 1780 Hz/pixel). Another acquisition with the same parameters, but with inverse phase encoding direction (PA) and 3 directions at *b* = 0 s/mm^2^ values, has been acquired for distortion correction purposes. Along with this standardized dMRI protocol, for all subjects, a 3D-T1-weighted volume was acquired on a sagittal plane with high resolution (voxel size ≤ 1mm^3^). A complete list of all the scanners used at each site, along with the sequence parameters for the 3D-T1-weighted volume, is available in Supplementary Materials.

### MRI data analysis

The 3D-T1-weighted images were segmented in (1) gray matter, (2) sub-cortical gray matter, (3) white matter, (4) cerebrospinal fluid, (5) eventual pathological tissue (absent in all cases) using the *5ttgen* command in MRtrix3 and the *fsl* algorithm [25, 26].

All dMRI images were denoised [27, 28] and pre-processed using the FMRIB Software Library (FSL version 6.0) toolbox (http://www.fmrib.ox.ac.uk/fsl), using the *eddy* and *topup* distortion correction commands [29–31]. Although a standardized sequence was acquired in all sites, to further remove any possible additional variance caused by site differences, the ComBat data harmonization method was used (https://github.com/Jfortin1/ComBatHarmonization [32]).

Diffusion tensor model was fitted for each subject using *b* ≤ 1000 s/mm^2^ data [33], with fractional anisotropy (FA), mean diffusivity (MD), and radial diffusivity (RD) maps that were computed from this model [34]. Along with this approach, the Neurite Orientation Dispersion and Density Imaging (NODDI) model [35] was also fitted (using the Accelerated Microstructure Imaging via Convex Optimization—AMICO—software [36]) to obtain the neurite density index (NDI) maps, that was then weighted by the tissue fraction (NDI_w_) to avoid any bias in the microstructural map [37].

All processing steps were controlled, and case-by-case reviewed by an experienced user, to ensure the quality of the output.

### Statistical analyses

#### Between group differences—clinico-demographic and global WM evaluation analyses

Possible differences in terms of age, sex, and clinical scores between patients and controls were tested via Wilcoxon rank sum and Pearson’s Chi-squared tests, respectively.

Between-groups differences (SPG7 vs. HC, and ARSACS vs. HC) in terms of global volume and mean WM microstructure were tested via a robust linear regression using age and sex as covariates (along with the total intracranial volume for the global volume analysis, to take into account for head size).

Furthermore, to investigate a possible role of genotype in determining the observed MRI phenotype of WM damage, an ancillary analysis was performed with the same approach comparing WM variables in patients with a different genetic profile.

All these analyses were performed using RStudio software (https://www.R-project.org), with a significance level set for *p* < 0.05.

#### Between group differences—voxel-wise analysis

Possible voxel-wise differences of the investigated microstructural measures (namely, FA, MD, RD, and NDI_w_) between the patients and controls (namely, ARSACS vs. HC and SPG7 vs. HC) were probed using the Tract-Based Spatial Statistics (TBSS) analysis [13]. In particular, each map was co-registered to the FMRIB58_FA template available in FSL, in the Montreal Neurological Institute (MNI) space, using FNIRT’s nonlinear registration tool [38]. Normalized FA maps, visually assessed to ensure good quality of the normalization, were then used to create a WM “skeleton” that represents the alignment-invariant tracts in common to all subjects, applying a thinning algorithm to the average FA map with a threshold set equal to 0.2 [13]. Finally, each subject’s microstructural maps were projected onto this skeleton for subsequent between-group analyses, all age and sex corrected, using the FSL’s *randomise* tool (number of permutations = 5000). All results were considered significant for *p* < 0.05. Corrected for multiple comparisons using a Threshold-Free Cluster Enhancement (TFCE) approach [13].

Finally, to evaluate the clinical counterparts of the observed microstructural changes in ARSACS and SPG7 compared to HC, possible correlations between FA values (as the main index of microstructural damage [39, 40], disease duration (calculated as the difference between age at onset and MR date) and clinical scores were also probed via voxel-wise analyses, using the same approach and a statistical threshold described before. In addition, analyses were also probed adding the WM volume as additional covariate, to test whether the possible observed correlations were influenced by WM macrostructural changes.

## Results

After application of inclusion and exclusion criteria, a final number of 103 subjects from 5 sites were included in this study, with MRI data of 37 ARSACS patients (M/F = 21/16, mean age = 33.4 ± 12.4 years), 37 SPG7 patients (M/F = 24/13, mean age = 55.7 ± 10.7 years), and 29 HC (M/F = 13/16, mean age = 42.1 ± 17.2 years) that were analyzed (Fig. 1). Demographic and clinical data of the subjects included in this study are available in Table 1, with a breakdown of these variables divided per site available in the Supplementary Table 1.

**Table 1:** Demographic and clinical data of the subjects included in this study

	ARSACS (*n* = 37)	SPG7 (*n* = 37)	HC (*n* = 29)	*p* value (ARSACS vs. HC)	*p* value (SPG7 vs. HC)
Age	34 [16–63]	56 [34–73]	41 [17–77]	0.04	0.001
Sex (M/F)	21/16	24/13	13/16	0.34	0.10
AAO	6.9 ± 7.9	36.8 ± 11.6	n.a	n.a	n.a
SARA	16 [4–33]	9.5 [2–23]	0 [0–4]	<0.001	<0.001
SPRS	24 [6–39]	16 [6–36]	0 [0–5]	<0.001	<0.001
fSPRS	15 [1–24]	10 [5–24]	0 [0–5]	<0.001	<0.001
FARS	23 [2–46]	20 [4–51]	0 [0–6]	<0.001	<0.001
MoCA	24 [15–29]	26 [14–29]	28 [22–30]	<0.001	<0.001

Age at onset is reported as mean and standard deviation, while age and clinical scores are reported as median values, with corresponding ranges in brackets

*AAO* age at onset, *ARSACS* Autosomal Recessive Spastic Ataxia of Charlevoix-Saguenay, *SPG7* Spastic Paraplegia-7, *HC* Healthy Controls, *SARA* Scale for the Assessment and Rating of Ataxia, *SPRS* Spastic Paraplegia Rating Scale, *fSPRS* “functional mobility” items from 1 to 6, *FARS* Friedreich’s Ataxia Rating Scale, *MoCA* Montreal Cognitive Assessment test

**Fig. 2:**
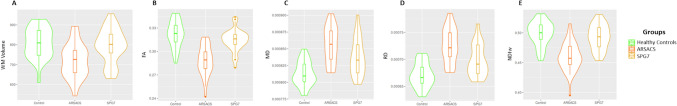
Violin plots showing the differences between the three groups in terms of WM volume (**A**) and global mean microstructural metrics (FA, MD, RD, and NDI_w_ in **B**–**E**, respectively). WM = white matter; FA = fractional anisotropy; MD = mean diffusivity; RD = radial diffusivity; NDI_w_ = neurite density index weighted by tissue fraction

When evaluating possible differences in terms of global WM metrics, ARSACS patients showed a significant reduction of WM volume compared to HC (719.6 ± 83.7 vs. 809.9 ± 75.9 mL, *p* < 0.001), while a similar but less pronounced difference that emerged when SPG7 patients and HC were compared (793.9 ± 84.2 vs. 809.9 ± 75.9 mL, *p* = 0.01) (Fig. 2A). Similarly, a different behavior between ARSACS and SPG7 patients was found for all mean microstructural WM metrics. Indeed, while ARSACS patients showed changes in all the dMRI-derived metrics probed (*p* < 0.001 for all dMRI metrics), SPG7 showed an increase in mean MD (0.83 ± 0.027 vs. 0.81 ± 0.018 × 10^−3^ mm^2^/s, *p* = 0.006) and RD (0.69 ± 0.029 vs. 0.67 ± 0.022 × 10^−3^ mm^2^/s, *p* = 0.01) values compared to HC, while no differences emerged for FA (*p* = 0.21) and NDI_w_ (*p* = 0.09) values. The ancillary analysis investigating the possible role of genetic profile in determining the observed MRI phenotype of WM damage showed no significant differences between subjects harboring one or two truncated alleles and those with a missense mutation of the affected gene (Supplementary Table 2).

A complete list of the results of the global WM evaluation analyses is available in Table 2, with a graphical representation shown in Fig. 2.

**Table 2:** Results of the global WM evaluation analyses

	ARSACS	SPG7	HC	*p* value (ARSACS vs. HC)	*p* value (SPG7 vs. HC)
WM volume	719.6 ± 83.7	793.9 ± 84.2	809.9 ± 75.9	<0.001	0.05
FA	0.29 ± 0.020	0.31 ± 0.013	0.32 ± 0.016	<0.001	0.21
MD	0.85 ± 0.018	0.83 ± 0.027	0.81 ± 0.018	<0.001	0.006
RD	0.73 ± 0.031	0.69 ± 0.029	0.67 ± 0.022	<0.001	0.01
NDI_w_	0.46 ± 0.023	0.49 ± 0.020	0.50 ± 0.020	<0.001	0.09

Volumes are reported in milliliters, MD and RD maps’ values are reported in 10^−3^ mm^2^/s, while FA and NDI_w_ are adimensional. All values are expressed as mean and standard deviation

*ARSACS* Autosomal Recessive Spastic Ataxia of Charlevoix-Saguenay, *SPG7* Spastic Paraplegia-7, *HC* Healthy Controls, *WM* white matter, *FA* fractional anisotropy, *MD* mean diffusivity, *RD* radial diffusivity, *NDI*_*w*_ neurite density index weighted by tissue fraction

**Fig. 3:**
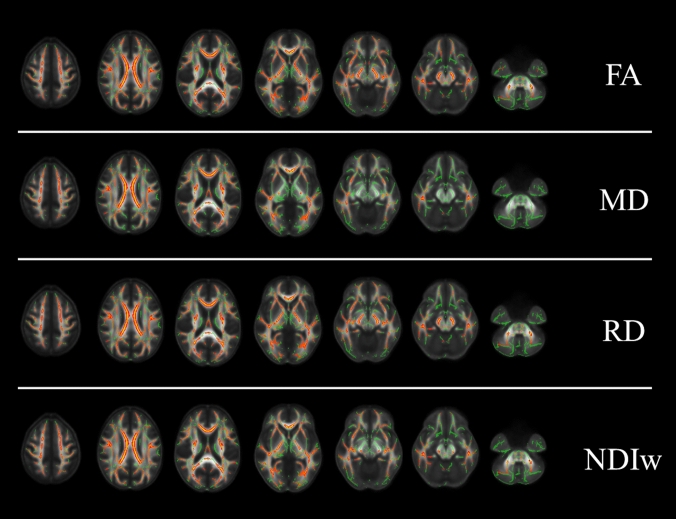
Results of the TBSS analysis showing the spatial distribution of microstructural damage in ARSACS patients. Significant differences, in a yellow–red color code scale superimposed to the WM “skeleton” in green, are shown for anatomic reference on selected axial slices of the FA template in the standard MNI space. TBSS = Tract-Based Spatial Statistics; MNI = Montreal Neurological Institute; FA = fractional anisotropy; MD = mean diffusivity; RD = radial diffusivity; NDI_w_ = neurite density index weighted by tissue fraction

When possible voxel-wise differences in terms of microstructural WM metrics between the three groups were evaluated, we found widespread changes affecting the WM of ARSACS patients, without a spatially distributed pattern of damage but with a severe involvement of commissural fibers (such as the corpus callosum and the fornix) and of the infratentorial structures (such as the pons and the middle cerebellar peduncles) (Fig. 3). In line with the results of the global WM analysis, SPG7 patients showed no significant voxel-wise differences in terms of WM microstructure compared to HC.

**Fig. 4:**
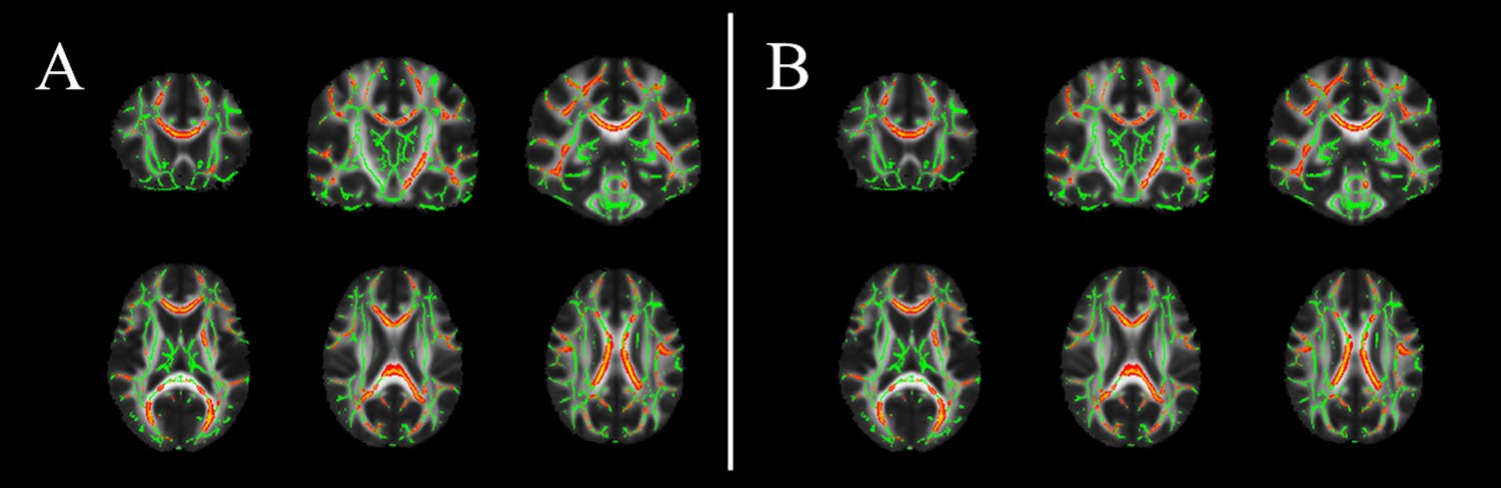
Results of the voxel-wise correlation analysis between microstructural integrity of the WM and clinical scores, showing the spatial distribution of correlation between FA values and SARA scores without (**A**) and with (**B**) correction for WM volume. Voxel of significant correlations, in a yellow–red color code scale superimposed to the WM “skeleton” in green, are shown for anatomic reference on the FA template in the standard MNI space in selected coronal (upper row) and axial (lower row) slices. MNI = Montreal Neurological Institute; FA = fractional anisotropy; WM = white matter; SARA = Scale for the Assessment and Rating of Ataxia

Finally, when possible correlations between clinical scores and WM microstructure were probed, we observed a negative correlation between SARA scores and FA values (*p* = 0.004) in ARSACS patients, with a more significant involvement of the commissural fibers, and in particular the genu of the corpus callosum (Fig. 4A). These results remained significant after correction for WM volume, suggesting their independence from atrophy (Fig. 4B). No significant correlation emerged between FA values and diseased duration (*p* = 0.22) or the remaining clinical scales (*p* = 0.14 and *p* = 0.64 for SPRS and FARS, respectively), with the exception of two borderline correlations with fSPRS and MoCA scores (*p* = 0.05 for both tests).

## Discussion

In this study, we performed a thorough evaluation of WM involvement in one of the largest groups of ARSACS and SPG7 patients using a standardized and harmonized MRI protocol among five different centers worldwide. Our results showed that while subjects with ARSACS present considerable WM involvement both at a macrostructural and microstructural level, SPG7 patients have relatively preserved WM macrostructure and microstructure.

With reference to the findings in ARSACS, we proved a severe macrostructural WM involvement (suggested by a global WM volume loss), coupled to profound and widespread microstructural changes (with changes in all dMRI-derived measures we evaluated). To date, most of the currently available dMRI literature in ARSACS has been focused on the evaluation, using diffusion tensor imaging (DTI), of possible corticospinal tract (CST) and pontine changes occurring in these patients [16, 41, 42]. Nonetheless, a similar approach only allows to evaluate some specific features of a condition, possibly neglecting additional meaningful information on other brain regions. This limitation is overcome using voxel-wise analyses, which are known to be highly reproducible, user-independent, and can explore differences over the entire brain without anatomically specific hypotheses [43]. The findings of our study are in line with data in a smaller group of ARSACS patients (*n* = 9) suggesting the possible occurrence of microstructural changes in some supratentorial and cerebellar areas, as well as through the CST [17]. Similarly, another dMRI study showed the presence of microstructural involvement of different commissural fibers (including forceps minor, forceps major and superior longitudinal fasciculum) in a different small group of patients, suggesting how structural changes might indeed extend beyond motor pathways, also involving the key associative fiber bundles [16]. Thanks to a significantly higher power size and multi-center involvement, our results expand this knowledge, showing the occurrence of multi-faced global neuronal involvement in ARSACS, with loss of neurites integrity, secondary demyelination and overall, a reduction in cellularity, without a specific spatial distribution but with a more prominent involvement of commissural fibers, including the corpus callosum. In particular, the reported loss of axonal and dendritic integrity (often referred together as neurites) is a novel finding in ARSACS. Changes in neurite organization was inferred by the observed changes in NDI, a metric derived from the NODDI model [35] which is known to be able to provide better explanations compared to “classic” dMRI models by increasing specificity for clinically meaningful tissue properties [44]. From a pathophysiological standpoint, this finding could be explained, at least in part, by the evidence of an alteration in mitochondrial transport and dendritic architecture reported in *sacsin* knockout mice, that seems to precede Purkinje cell death and neurodegeneration [45]. Furthermore, it has also been speculated that *sacsin* might have a role in the development phase of the brain [16], with a possible imaging counterpart of this abnormal neurodevelopment that could be observed in the misplacement of pontine fibers reported in ARSACS patients[17, 41, 42] as possible sign of aberration of axonal guidance in this condition[46].

Along with modifications in axonal and dendritic integrity, we also found changes in FA, MD, and RD metrics. While FA and MD provide sensitive but non-specific measures of pathology that may be affected by different factors [47], RD changes are supposedly linked to secondary demyelination phenomena. A possible explanation for these observed RD changes can be researched in glial involvement in ARSACS, given the reported accumulation of astroglial intermediate filaments, including glial fibrillary acidic protein (GFAP), in a *sacsin* knockout rodent model [48]. As astrocytes are known to participate in neuroinflammation [49] and establish numerous interactions with other cells in the nervous system, including neurons [50], we speculate that the observed RD changes (a putative marker of demyelination) might be, therefore, linked to glial involvement, although future research is warranted to elucidate the mechanisms behind this possible link. Interestingly, the voxel-wise analysis showed how commissural fibers, and in particular the corpus callosum, seem to be predominantly involved in ARSACS, also showing a degree of correlation with SARA scores, in absence of correlation with disease duration (thus suggesting that WM changes might represent an early phenomenon in the pathophysiology of damage in these patients). The involvement of this major WM tract is not unexpected, given the reported *sacsin* expression in this structure [51], which could have a “macroscopical” reflection in the (qualitatively evaluated) reported volume loss of this region [52]. Taken together, our results suggest a prominent role of the corpus callosum in ARSACS, advising for future specific investigations of this major commissural tract, also to evaluate if possible modifications over time of its microstructure could serve as potential biomarker of the disease.

Despite the neuropathologic hallmark of Hereditary Spastic Paraplegias is considered the length-dependent distal axonal degeneration of the CST [53], and that a reduced WM integrity of this structure (along with the brainstem and the frontal lobes) has been anecdotally reported in SPG7 [18, 19], we failed to find any significant microstructural WM change in our group of patients. This suggests that if brain WM microstructure is relatively preserved in SPG7, other neuronal mechanisms of damage might be present, such as a preferential involvement of long axons of peripheral nervous systems. Supporting this speculation, in SPG7 knockout mice, a subset of mitochondria has been selectively reported in the distal region of axons in the spinal cord and the sciatic nerves [54]. Nonetheless, the mechanisms underlying axonal damage require future research.

Although this study has some strengths, such as the large sample size, the acquisition of a standardized harmonized MR protocol across all centers, and the application of advanced analysis models such as NODDI, some limitations should be discussed. In particular, dMRI is not the only technique that can be used to evaluate WM microstructure using MR, with other information that can be obtained by evaluating, for instance, Magnetization Transfer Ratio or Relaxometry. Nonetheless, dMRI is widely accepted as a valuable tool to comprehensively evaluate microstructure in the field of ataxias [5]. Furthermore, a longitudinal evaluation of these variables is also warranted, to understand their possible changes over time, and therefore to fully establish a possible role of dMRI-derived metrics as reliable imaging biomarker in these conditions. Finally, this study was focused on detecting whole brain WM abnormalities, reducing our sensitivity in the detection of possible changes that might occur in specific, small but relevant WM bundles unfeasible to be properly investigates via TBSS (i.e., the superior or inferior cerebellar peduncles) or disregarding microstructural information of other areas of the CNS, such as the spinal cord. Future studies are warranted to investigate the presence of other possible significant WM abnormalities affecting specific WM tracts and the spinal cord in these patients.

Despite the limitations, in this study, we showed how ARSACS, but not SPG7 patients, present a significant, complex and multi-faced involvement of brain WM, with a clinically meaningful widespread loss of axonal and dendritic integrity, secondary demyelination and, overall, a reduction in cellularity and volume.

### Supplementary Information

Below is the link to the electronic supplementary material.

Supplementary file1 (DOCX 29 KB)

## Data Availability

The data that support the findings of this study are available from the corresponding author upon reasonable request.

## Abbreviations

ARSACS: Autosomal Recessive Spastic Ataxia of Charlevoix-Saguenay
FARS: Friedreich’s Ataxia Rating Scale
SARA: Scale for the Assessment and Rating of Ataxia
SPAX: Spastic ataxia
SPG7: Hereditary Spastic Paraplegia Type 7
SPRS: Spastic Paraplegia Rating Scale
