# Supplementary material for: An MRI evaluation of white matter involvement in paradigmatic forms of spastic ataxia: results from the multi-center PROSPAX study
Source: J Neurol. 2024 Jun 16;271(8):5468–77. doi: 10.1007/s00415-024-12505-y (PMC11319608; doi:10.1007/s00415-024-12505-y)
Supplement: Supplementary file 1 — Supplementary file1 (DOCX 29 KB) [file 415_2024_12505_MOESM1_ESM.docx]

**MATERIAL AND METHODS**

*MRI data acquisition and analysis*

All MRI brain scans were performed at 3T, with the acquisition protocol that included, for all sites, a standardized diffusion sequence (see main text for the parameters) and a whole brain 3D-T1-weighted sagittal sequence. For all sites scanner name and main image parameters are here listed:

- Site 1 (Essen, Germany), Siemens Vida (Siemens Medical Systems, Erlangen, Germany), Magnetization-Prepared Rapid Acquisition with Gradient Echo (MPRAGE) sequence, TR=2900ms, TE=3.10ms, TI=1030ms, FA= 9°, voxel size=0.8×0.8×0.8mm^3^, 208 slices;

- Site 2 (Montreal, Canada), Siemens Prisma (Siemens Medical Systems, Erlangen, Germany), MPRAGE sequence, TR= 2300ms, TE= 2.38ms, TI= 900ms, FA= 8°, voxel size= 0.8x0.8x0.8mm^3^, 192 slices;

- Site 3 (Naples, Italy), Siemens Trio (Siemens Medical Systems, Erlangen, Germany), MPRAGE sequence, TR= 2400ms, TE= 2.12ms, TI=1000ms, FA= 8°, voxel size= 0.8×0.8×0.8mm^3^, 208 slices;

- Site 4 (Nijmegen, Netherlands), Siemens Prisma (Siemens Medical Systems, Erlangen, Germany), MPRAGE sequence, TR=2300ms, TE=2.32ms, TI=900ms, FA=8°, voxel size=0.9x0.9x0.9mm^3^, 192 slices;

- Site 5 (Tübingen, Germany), Siemens Vida (Siemens Medical Systems, Erlangen, Germany), MPRAGE sequence, TR=2900ms, TE=3.13ms, TI=900ms, FA=9°, voxel size=0.8×0.8×0.8mm^3^, 208 slices.

**RESULTS**

A complete list of all demographic and clinical variables divided per site is reported in Supplementary Table 1.

**SUPPLEMETARY TABLES**

**SUPPLEMETARY TABLE 1**

| **Site 1 – Essen, Germany** | | | | | | |
| --- | --- | --- | --- | --- | --- | --- |
|  | | | *ARSACS*  *(n=1)* | | *SPG7*  *(n=8)* | *HC*  *(n=5)* |
| Age | | | 63 | | 54.8 ± 11.3 | 47 ± 21.5 |
| Sex (M/F) | | | 0/1 | | 4/4 | 3/2 |
| AAO | | | 9 | | 38.5±14.6 | n.a. |
| SARA | | | 33 | | 10 [6 – 23] | 0 [0 – 1] |
| SPRS | | | 32 | | 18 [12 – 31] | 0 [0 – 2] |
| fSPRS | | | 24 | | 11 [8 – 23] | 0 [0 – 2] |
| FARS | | | 22 | | 21.5 [8 – 33] | 0 |
| **Site 2 – Montreal, Canada** | | | | | | |
|  | | | *ARSACS*  *(n=12)* | | *SPG7*  *(n=4)* | *HC*  *(n=5)* |
| Age | | | 32.3±7.7 | | 52.8±4.6 | 31 ± 10.1 |
| Sex (M/F) | | | 7/5 | | 3/1 | 4/1 |
| AAO | | | 2.7±1.7 | | 34.5±6.7 | n.a. |
| SARA | | | 16.5 [11 – 30] | | 13 [9 – 17] | 0 |
| SPRS | | | 25 [8 – 39] | | 27 [10 – 28] | 0 |
| fSPRS | | | 14.5 [5 – 24] | | 15.5 [6 – 17] | 0 |
| FARS | | | 24 [10 – 46] | | 28 [13 – 34] | 0 |
| **Site 3 – Naples, Italy** | | | | | | |
|  | | *ARSACS*  *(n=5)* | | *SPG7*  *(n=4)* | | *HC*  *(n=7)* |
| Age | | 41.6±9.5 | | 63.3±15.0 | | 36.1 ± 18.1 |
| Sex (M/F) | | 3/2 | | 3/1 | | 2/5 |
| AAO | | 10.2±14.9 | | 37.0±9.6 | | n.a. |
| SARA | | 14 [11 – 17] | | 17.5 [9 – 23] | | 0 [0 – 3] |
| SPRS | | 25 [18 – 33] | | 29.5 [14 – 36] | | 0 [0 – 5] |
| fSPRS | | 16 [10 – 18] | | 19.5 [9 – 24] | | 0 [0 – 3] |
| FARS | | 23 [9 – 35] | | 26.5 [13 – 51] | | 0 |
| **Site 4 – Nijmegen, Netherlands** | | | | | | |
|  | | *ARSACS*  *(n=4)* | | *SPG7*  *(n=9)* | | *HC*  *(n=5)* |
| Age | | 33.3±12.1 | | 56.8±8.4 | | 40±13.2 |
| Sex (M/F) | | 1/3 | | 6/3 | | 2/3 |
| AAO | | 10.3±8.5 | | 40.7±12.4 | | n.a. |
| SARA | | 22 [9 – 25] | | 9 [2 – 19] | | 0.5 [0.5 – 1] |
| SPRS | | 22.5 [9 – 38] | | 14 [6 – 25] | | 1 [0 – 5] |
| fSPRS | | 13.5 [6 – 23] | | 9 [5 – 17] | | 1 [0 – 5] |
| FARS | | 12 [6 – 30] | | 16 [4 – 31] | | 0 |
| **Site 5 – Tübingen, Germany** | | | | | | |
|  | *ARSACS*  *(n=15)* | | | *SPG7*  *(n=12)* | | *HC*  *(n=7)* |
| Age | 29.5 ± 13.6 | | | 54.1 ± 12.1 | | 54.1 ± 15.0 |
| Sex (M/F) | 10/5 | | | 8/4 | | 2/5 |
| AAO | 7.4±6.8 | | | 33.3±11.3 | | n.a. |
| SARA | 16 [4 – 24] | | | 9 [6 – 12] | | 0.5 [0 – 4] |
| SPRS | 20 [6 – 37] | | | 15.5 [8 – 27] | | 0 [0 – 2] |
| fSPRS | 11 [1 – 24] | | | 9 [5 – 17] | | 0 [0 – 2] |
| FARS | 17 [2 – 37] | | | 15.5 [8 – 39] | | 0 [0 – 1] |

Age is reported as mean and standard deviation, while clinical scores are reported as median values.

The corresponding ranges are reported in brackets**.**

AAO *= Age at onset; FARS = Friedreich's Ataxia Rating Scale; SARA = Scale for the Assessment and Rating of Ataxia; SPRS = Spastic Paraplegia Rating Scale*

**SUPPLEMETARY TABLE 2:** Results of the global WM evaluation analyses in ARSACS patients after stratification for allele truncation

|  | **ARSACS Group 0**  **(n = 2)** | **ARSACS Group 1**  **(n = 8)** | **ARSACS Group 2**  **(n = 27)** | **p-value**  (Groups 0 vs 1) | **p-value**  (Groups 0 vs 2) | **p-value**  (Groups 1 vs 2) |
| --- | --- | --- | --- | --- | --- | --- |
| *WM volume* | 668.3±127.5 | 687.1±60.5 | 733.0±85.9 | 0.70 | 0.46 | 0.15 |
| *FA* | 0.29±0.02 | 0.29±0.02 | 0.29±0.02 | 0.90 | 0.70 | 0.80 |
| *MD* | 0.84±0.03 | 0.85±0.03 | 0.86±0.02 | 1 | 0.21 | 0.30 |
| *RD* | 0.71±0.009 | 0.72±0.04 | 0.73±0.03 | 1 | 0.28 | 0.44 |
| *NDIw* | 0.46±0.002 | 0.47±0.02 | 0.46±0.02 | 1 | 0.41 | 0.07 |

Group 0 include patients without truncated alleles (only missense mutations), while Groups 1 and 2 include patients with one or two truncated alleles, respectively.

Volumes are reported in milliliters, MD and RD maps’ values are reported in 10^-3^ mm^2^/s, while FA and NDIw are adimensional. All values are expressed as mean and standard deviation.

*ARSACS = Autosomal Recessive Spastic Ataxia of Charlevoix-Saguenay; SPG7 = Spastic Paraplegia-7; HC = Healthy Controls; WM = white matter; FA = fractional anisotropy; MD = mean diffusivity; RD = radial diffusivity; NDIw = neurite density index weighted by tissue fraction.*
